# Mechanotransduction in breast cancer and implications for preclinical modeling

**DOI:** 10.1007/s10555-026-10396-1

**Published:** 2026-11-10

**Authors:** Matthew D. Harrington, Jinghui Sun, Grayson Whitten, Hai Yao, Heather W. Dunn

**Affiliations:** 1 Department of Bioengineering, Clemson University, Clemson, SC USA; 2 Department of Bioengineering, Clemson University at the Medical University of South Carolina, Charleston, SC USA

**Keywords:** Mechanotransduction, Breast cancer, Extracellular matrix, Tumor microenvironment, Preclinical models, Health disparities

## Abstract

Breast cancer remains a leading cause of cancer morbidity and mortality, representing the most frequently diagnosed new cancer of 2024. Despite overall improvements in survival, women of African ancestry experience disproportionately higher rates of aggressive subtypes and adverse outcomes, signifying persistent biological and clinical gaps. Mechanotransduction, the process by which cells sense and respond to mechanical cues, regulates mammary homeostasis, tumor progression, metastatic dissemination, and therapeutic response. However, its context-dependent and bidirectional roles remain incompletely resolved, and reproducing biomechanical environments in preclinical models remains a substantial challenge. This review synthesizes current understandings of canonical and cancer-associated mechanotransduction in breast tissue, including cell–extracellular matrix (ECM) and cell–cell adhesions, mechanosensitive ion channels, glycocalyx remodeling, cytoskeletal dynamics, nuclear force transmission, and mechanosensitive transcription. It further examines how ECM stiffness, viscoelasticity, ligand availability, collagen architecture, tensile and compressive stresses, and stromal and immune cell activity influence invasion, metastasis, and treatment resistance. Ancestry-associated stromal, molecular, and structural variations are considered as a critical motivation, but remain incompletely characterized as sources of ancestry-dependent heterogeneity. Thus, this review explores mechanotransduction as a framework for assessing proliferation and metastasis mechanisms through two-dimensional culture, three-dimensional matrices, spheroids, organoids, co-cultures, bioreactors, organ-on-a-chip systems, bioprinting, animal models, and computational approaches for their capacity to reproduce these processes. We ultimately propose an iterative design–build–validate framework that links empirically derived mechanical and molecular measurements to model selection, construction, and multilevel validation. Integrating mechanobiological heterogeneity into rigorously benchmarked models could improve understanding of metastatic progression, reveal context-dependent therapeutic resistance, and support more representative and patient-informed breast cancer research.

## Introduction

The earliest known records of breast cancer date back to ancient Egypt, circa 5000 years ago, documented in what is now known as the Edwin Smith Papyrus. Physician Imhotep describes the diagnosis of breast tumors, citing tissue stiffness as a key characteristic of the disease [1]. In the 2020 s, breast cancer continues to pose a critical public health challenge, representing the most common new cancer diagnosis in 2024 [2]. Although advances in tumor biology have led to improved survival rates, breast cancer initiation and progression mechanisms remain incompletely resolved. Increasingly, an understanding of the role of aberrant mechanotransduction, the process by which cells convert mechanical cues from their environment into biochemical signals, in breast cancer has increased. This process is bidirectional: cells not only sense physical forces, but also reciprocally remodel their microenvironment through cytoskeletal contractility and matrix deposition, creating a feedback loop that drives tumor progression [3–5]. In the human mammary gland, tissue stiffness, extracellular matrix (ECM) remodeling, and altered cellular tension profoundly influence oncogenic signaling, metastatic potential, and therapeutic resistance [6]. Dysregulated mechanotransductive pathways involving integrins, focal adhesion kinases (FAK), or YAP/TAZ transcriptional regulators, among others, not only sustain malignant phenotypes but also contribute to tumor heterogeneity and microenvironmental adaptation [7]. Thus, further characterizing and accurately modeling these biomechanical signaling networks is essential for elucidating the complex interplay between the tumor and the mechanics of its environment.

**Fig. 1:**
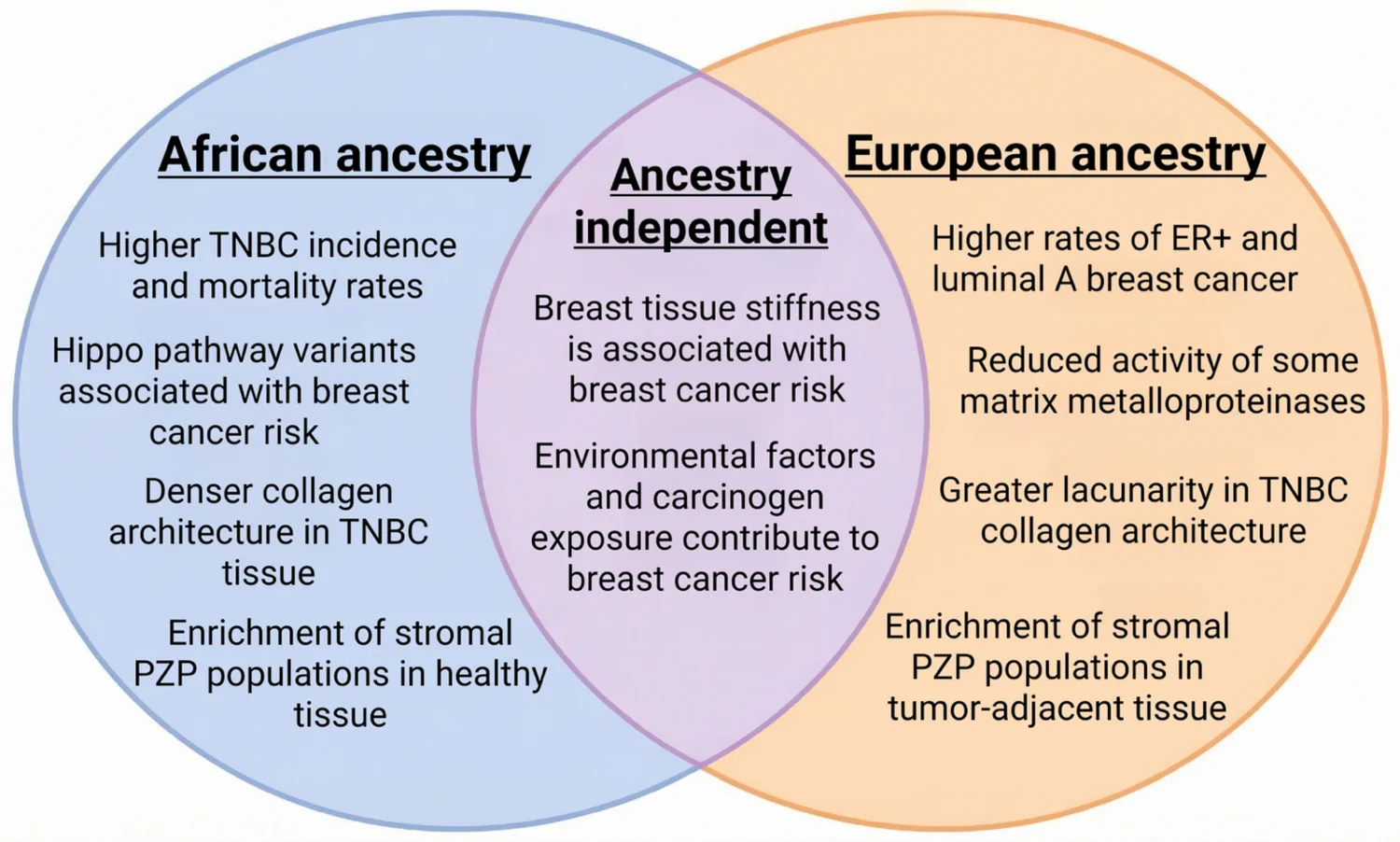
Ancestry-associated features relevant to breast cancer mechanobiology. Venn diagram summarizing selected genetic, molecular, epidemiologic, stromal, and tissue-structural features reported in association with ancestry or self-identified race or ethnicity in breast cancer. The central region identifies general mechanobiological features of breast cancer that have not been robustly implicated as ancestry-specific. Entries on either side illustrate relevant factors that have been implicated in contributing to breast cancer disparities along the gradient of patient ancestry. Individual entries are not interpreted as uniformly differentially expressed across all members of a population or as singular causal determinants of clinical outcomes. [2, 10, 11, 13–20]. Figure created with BioRender

Research has demonstrated that stiffer breast tissue increases the rates of epithelial-to-mesenchymal transition (EMT) via mechanotransduction mechanisms, including TWIST1 and G3BP2 [8]. Further, nontumorigenic stromal cells in the microenvironment can also remodel the ECM into a more tumor-permissive and prometastatic state [9]. However, the risks of aberrant mechanotransduction promoting more severe and invasive forms of breast cancer are not the same for all patients. Women of African ancestry often exhibit distinct physiological and molecular patterns in breast tissue that may contribute to a higher risk of aggressive breast cancer subtypes and poorer clinical outcomes [10, 11]. Epidemiologic studies consistently show that women of African ancestry have disproportionately higher incidence of triple-negative breast cancer (TNBC), a subtype with limited targeted therapy options and poor prognoses, compared with women of European ancestry [2]. Transcriptomic profiling has revealed ancestry-associated gene expression differences in both normal breast tissues and TNBC tumors from women of African descent that correlate with altered tumor biology and immune microenvironments, suggesting that intrinsic cellular programs differ by ancestry [12]. Further research also indicates that distinct stromal cell populations, such as those overexpressing PROCR, ZEB1, and PDGFRa (PZP) in healthy breast tissue of women of African ancestry, may influence epithelial invasion and mechanobiological behaviours relevant to cancer progression [13]. While these transcriptomic differences are well-documented, their impact on the biomechanical landscape remains underappreciated. Crucially, these ancestry-associated stromal populations may drive a distinct mechanical niche characterized by altered collagen alignment or increased stiffness, thereby priming the tissue for aggressive outcomes via enhanced mechanotransduction (Fig. 1).

Despite major advances in molecular oncology, conventional two-dimensional (2D) culture systems and assorted animal models often fail to recapitulate the complex mechanical heterogeneity of human breast tumors. Standard 2D monolayer cultures lack key biophysical cues inherent to the *in vivo* setting, such as fluid shear stress, interstitial fluid pressure, and three-dimensional (3D) stiffness gradients [21, 22]. This limitation has driven the development of increasingly sophisticated preclinical models designed to capture mechanotransductive dynamics more faithfully. 3D spheroids and organoids enable the study of cell–ECM interactions and force-mediated morphogenesis *in vitro* [23], while bioreactors and organ-on-a-chip technologies introduce mechanical cues, such as shear stress and matrix stiffness, in more physiologically relevant contexts [24, 25]. Complementary *in vivo* approaches, including the implementation of patient-derived scaffolds and engineered animal models, offer valuable insights into tumor–stroma crosstalk and mechanical remodeling within the architecture of native tissue [26].

As preclinical systems continue to evolve, the integration of machine learning and artificial intelligence (AI)-driven analytical frameworks offers new opportunities to bridge the gap between mechanobiology and translational oncology. AI and machine learning algorithms can identify biomechanical biomarkers and predict tumor behavior from complex mechanical datasets [27]. Together, these technologies are poised to refine our understanding of mechanotransduction in breast cancer and enhance the predictive power of preclinical modeling. This review critically examines current insights and new developments in mechanotransductive signaling in breast cancer biology and evaluates emerging model systems that seek to replicate its mechanical and biological complexity.

## Breast cancer mechanobiology

### Canonical mechanotransduction in healthy breast tissue

In healthy mammary tissue, cells continuously engage in mechanosensing and mechanotransduction to monitor and respond to their physical microenvironment, ensuring proper tissue architecture, function, and homeostasis [28]. The process of mechanosensing involves both cell–ECM and cell–cell adhesion systems, including integrin-based focal adhesions and cadherin-based adherens junctions, which couple mechanical cues to the cytoskeleton [29, 30].

Once mechanical cues are detected, they are converted into intracellular biochemical signals through interconnected pathways, including Hippo signaling [31], mechanosensitive ion channels, such as Piezo1 [32, 33], and adhesion- and cytoskeleton-dependent signaling involving talin unfolding, FAK activation [34], and Rho-family GTPase-regulated actin remodeling [35]. Mechanoresponsive transcriptional outputs include the myocardin-related transcription factor/serum response factor (MRTF/SRF) axis [35], NF-κB [36], β-catenin-dependent programs, and YAP/TAZ [37, 38]. These regulators contribute to changes in gene expression, cytoskeletal organization, proliferation, and differentiation. Preservation of this mechanical niche is critical for homeostatic function, yet distant variations in collagen fiber alignment can alter how forces are transmitted from the microenvironment to the cells [39]. Even during development, mammary epithelial cells cultured in soft 3D collagen gels utilize angular momentum to induce aligned acinar morphogenesis and milk protein secretion, processes dependent not only on biochemical cues, but also on a compliant mechanical environment [40]. Similarly, the repetitive stochastic forces cells experience during lactation correspond to increased Piezo1 expression, implicating mechanosensitive ion channels in the fundamental function of the healthy mammary gland [41].

At the molecular level, several interconnected pathways govern the mechanosensitive behaviour of mammary epithelial cells. Integrin engagement transmits force through talin and vinculin to the actin cytoskeleton, activating FAK/Src and Rho-family GTPases [28]. RhoA promotes stress fiber assembly, while Rac1 and Cdc42 promote lamellipodia and filopodia formation, respectively [42]. Polymerization of monomeric G-actin into filamentous F-actin releases MRTF-A from G-actin-mediated sequestration, permitting its nuclear accumulation and co-activation of the DNA-binding transcription factor SRF [35]. In human MCF10A acini, increased matrix stiffness was associated with higher MRTF/SRF reporter activity and increased protrusion formation [43]. Stiffness-associated protrusion formation was also observed in primary murine mammary organoids. Treatment with the Rho/MRTF/SRF pathway inhibitor CCG-203971 reduced protrusion formation in MCF10A acini under selected matrix conditions, whereas primary murine organoids showed a trend toward reduced protrusion formation at intermediate matrix stiffness [43].

Crucially, force transmission extends beyond the cytoskeleton to the nucleus via the linker of the nucleoskeleton and cytoskeleton (LINC) complexes [44, 45]. This structure, composed of SUN-domain proteins and KASH-domain-containing nesprins, physically couples the cytoskeleton to the nuclear lamina, thereby directly regulating chromatin organization and gene accessibility in response to mechanical stress [45–47].

Mechanically activated cation channels provide a mechanosensory route alongside integrin-based signaling. Mechanical deformation can activate Piezo channels and generate rapid cation influx, including Ca^2+^ influx [32, 48]. In mice, Piezo1 transcript levels increase in mammary tissue during lactation, and functional expression has been demonstrated in luminal epithelial cells. However, luminal-specific Piezo1 deletion did not measurably impair lactation or involution under the conditions examined [41]. Piezo channels provide a direct membrane-mechanical gating route, whereas TRPV4-dependent mechanosensitivity in mammary epithelium is coupled to tension, matrix stiffness, epithelial junction integrity, and pathways upstream of actomyosin contraction [49]. Critically, these channels would best be interpreted, then, according to their specific gating and cellular contexts, rather than as a uniform response to force.

On the cell surface, the bulky glycoprotein layer, known as the glycocalyx, acts as a primary mechanosensor for fluid shear stress [50, 51]. The physical bulk of this layer can also mechanically drive receptor organization, modulating integrin clustering and downstream signaling, even independent of matrix stiffness [51]. In mammary epithelial cells, appropriate ECM stiffness supports normal signaling via these pathways to maintain differentiation and tissue architecture, for instance, through the tuning of Rho-mediated contractility to ECM compliance [4]. These canonical systems provide the mechanobiological basis for how breast tissue senses and responds to its mechanical environment under healthy conditions.

Cell–cell junctions provide a complementary route for force sensing and transmission in mammary epithelium. Classical cadherins couple neighboring cells to cortical actin through β-catenin and α-catenin; tension-induced conformational changes in α-catenin recruit vinculin and reinforce adherens junctions [30]. In MCF10A mammary epithelial monolayers, systematic perturbation of the intercellular adhesome showed that cadherins and catenins regulate both intercellular force and collective cell dynamics: P-cadherin abundance predicted force magnitude, whereas E-cadherin abundance predicted the rate of force buildup [52]. Mammary epithelial cell lines also demonstrate mechanosensitive E-cadherin signaling in response to applied force. Flow-induced force applied to E-cadherin can activate LKB1-dependent AMPK signaling, increase glucose uptake and ATP production, and reinforce cell–cell junctions [53]. Thus, mammary epithelial mechanics depend not only on traction at cell–ECM adhesions, but also on force propagation and coordination across cell–cell junctions.

Mechanotransduction in the normal breast microenvironment also relies on dynamic reciprocity: cells not only sense and respond to mechanical cues but also actively remodel their ECM and adjust their contractile behaviour to maintain mechanical homeostasis [54]. Mammary gland development involves changes in ECM composition and organization, collagen, fibronectin, and laminin, that progressively tune matrix stiffness and architecture during branching morphogenesis and alveologenesis [55]. The tissue mechanics are thus regulated, at least in part, by epithelial–stromal interactions that balance matrix deposition, crosslinking, and cellular traction. If this equilibrium is perturbed, the mechanotransductive elements may trigger compensatory responses via cytoskeletal reorganization or ECM remodeling [28].

Faithfully recapitulating the healthy mechanotransductive environment of breast tissue is foundational to interpreting how aberrant phenotypes lead to pathology. Models that neglect to mimic relevant factors such as ECM stiffness, mechanosensor–cytoskeleton coupling, and ECM remodeling capacity risk misrepresenting baseline mechanotransduction. Analyses from the African American Breast Cancer Epidemiology and Risk (AMBER) Consortium identified associations between genetic variations in the Hippo pathway and breast cancer risk in women identified as African American [10]. However, whether these variants alter cellular responses to mechanical cues was not examined in that study. Thus, healthy models without representative, population-specific consideration of biomechanical signalling may mask or distort conclusions regarding how mammary epithelial cells respond to and initiate altered biomechanical conditions.

### Cancer-associated mechanotransduction

Normal and malignant mammary epithelial cells use many of the same mechanosensory components, but differ in how mechanical inputs are integrated with ECM composition, how long responses persist, and which cell state programs follow. In nonmalignant MCF10A acini, increasing ECM stiffness alone induced malignant features, whereas a parallel increase in basement-membrane ligands abrogated this effect; the combined stiffness-composition response involved β4 integrin, Rac1, and PI3K signaling [56]. During tumor progression, these contextual controls can be weakened or rewired, with increased integrin-FAK signaling, RhoA/ROCK activity, and myosin II-dependent contractility reinforcing traction generation, ECM remodeling, and invasive behaviour [6, 57]. Mechanosensitive ion channels provide a parallel example: Piezo1 has been implicated in normal mammary responses during lactation and involution [33], whereas tumor-associated compressive stress can activate a Piezo1/Ca^2+^/CaMKII/RhoA-ROCK pathway and establish epigenetic mechanical memory that promotes proliferation [58]. Further, TRPV4-mediated Ca^2+^ signaling has been shown to activate AKT, reduce E-cadherin expression, and promote transendothelial migration while contributing to cell softness [59].

While calcium channel outputs are themselves variable, their mechanoresponsive effects extend far beyond traditional YAP/TAZ activation. In metastatic breast cancer cells, Lee et al. demonstrated that TRPV4-dependent Ca^2+^ influx activated AKT and reduced E-cadherin expression [59]. Whereas, in mammary epithelial cells, a separate study found that TRPV4 depletion altered CaMKII and myosin light-chain phosphorylation, reduced force production, and disrupted junctional organization [49]. Azimi et al. further demonstrated the context-dependent outputs of TRPV4 by demonstrating that pharmacological TRPV4 activation induced EMT-associated events and markers. In the same study, TRPV4 knockdown reduced EGF- and hypoxia-induced vimentin expression, decreasing single-cell migration of MDA-MB-468 cells through a collagen type I gel [60]. Critically, calcium-dependent effects on actin organization, adhesion, and myosin-dependent contractility should therefore be considered as highly context-dependent, rather than uniformly activating.

Remodeling of the cell surface glycocalyx can further alter integrin-mediated mechanosignaling in mammary epithelial cells. High levels of the bulky glycoprotein MUC1 were detected in breast cancer cell lines and oncogene-transformed mammary epithelial cells. Expressing MUC1 at comparable levels in nontransformed mammary epithelial cells promoted integrin clustering and focal adhesion assembly, enhanced integrin-dependent growth-factor signaling, and supported growth and survival on compliant matrices [61]. These findings link tumor-associated changes in glycocalyx architecture to altered cell–ECM adhesion and growth factor responsiveness in mammary epithelial cells. Computational modeling further suggests that a bulky glycocalyx can promote force-dependent integrin catch bonding during early adhesion formation [62]. These integrin bonds are initially strengthened by applied tension or compression but are counterintuitively weakened when that force exceeds a given threshold. Choi et al. illustrated that these instances of mechanobiological coupling by integrins, particularly β1 integrins in developing thymocytes, are deactivated upon sema3E binding to plexinD1, a recently established modulator of cancer cell motility [63, 64].

A further distinction concerns how normal and malignant mammary epithelia respond over time to the same ECM environment. In 3D culture, collagen-I-induced protrusions and dissemination were observed in both normal and tumor mammary organoids. In normal organoids, dissemination was transient and ceased as laminin-111 accumulated along the basal surface; in tumor organoids, dissemination persisted, and basement membrane coverage remained incomplete [65]. Furthering this mechanistic distinction, Sharma et al. describe how oscillatory mechanical strain on TNBC cells induces overexpression of TGF-β/SMAD signaling, ultimately promoting tumor-permissive phenotypes and EMT [66]. These findings suggest that response termination and restoration of basement membrane organization distinguish normal from malignant epithelium, and further connect extracellular strain to increased mesenchymal behaviour.

Changes in cell–cell adhesion should also be considered alongside altered cell–ECM sensing. Cadherin–catenin complexes couple neighboring cells to the actin cytoskeleton [30], while perturbation of the intercellular adhesome in MCF10A monolayers alters intercellular tension and collective cell dynamics [52]. In nontumorigenic MCF10A cells, CDH1 deletion altered actin and microtubule organization and weakened cell–substrate adhesion, but the cells retained cell–cell contacts and did not undergo a complete EMT [67]. Thus, E-cadherin loss alone does not necessarily produce a complete EMT; junctional composition and cytoskeletal organization should be considered together when interpreting epithelial plasticity.

Across breast cancer models, E-cadherin loss can have different consequences for local invasion and later metastatic steps, according to comprehensive work by Padmanaban et al. In mouse luminal and basal invasive ductal carcinoma models, E-cadherin loss increased local invasion and dissemination but reduced circulating tumor cell numbers and metastasis. Additional experiments in the luminal model demonstrated impaired cancer cell survival, early lung seeding, and metastatic outgrowth [68]. In organoids derived from a human triple-negative breast cancer patient-derived xenograft in the same work, E-cadherin knockdown increased invasion and dissemination, but reduced colony formation; whereas E-cadherin-retaining mouse tumor organoid controls invaded collectively into collagen I [68]. These findings indicate that E-cadherin can restrain local invasion while supporting cancer cell survival and metastatic colonization.

Further dysregulation extends to Rho-family GTPases, which govern distinct aspects of cytoskeletal organization. RhoA promotes stress fiber assembly, while Rac1 and Cdc42 promote lamellipodia and filopodia formation, respectively [42]. In breast tumor models, increased matrix stiffness promoted 3D collective invasion through periostin-integrin signaling involving FAK, ERK, Cdc42, and Rac1, together with multicellular cytoskeletal remodeling [69]. These findings connect matrix-dependent signaling with the coordination of protrusion formation, adhesion remodeling, and collective invasion.

Crucially, global mechanoresponsive transcription in breast cancer is not restricted to YAP/TAZ activation alone. Although YAP and TAZ are prominent transcriptional co-activators, their contribution can vary with disease context and experimental models. Stiff substrates promoted YAP nuclear localization in 2D cultures of MCF10A mammary epithelial cells [70]. In contrast, YAP activation was not detected in the ductal carcinoma *in situ* specimens examined, and stiffness-induced proliferation and invasive phenotypes in mechanically tunable 3D MCF10A cultures occurred without corresponding YAP activation [70].

The Rho-actin-MRTF/SRF axis provides another route from cytoskeletal dynamics to transcription. In a study examining both MDA-MB-231 TNBC cells and B16F2 melanoma cells, depletion of MRTFs or SRF reduced adhesion, spreading, motility, and invasion and impaired experimental lung colonization [71]. β-catenin, which acts as a transcriptional co-regulator with TCF/LEF factors, is also mechanically responsive. In 2D cell cultures, stiff substrates promoted nuclear accumulation of YAP and β-catenin in MDA-MB-231 cells, whereas MCF-7 cells showed no significant stiffness-dependent redistribution of either protein. On soft substrates, YAP depletion in MDA-MB-231 cells increased β-catenin nuclear localization but reduced AXIN2 protein expression; these effects were not observed on stiff substrates [38].

NF-κB provides an additional mechanosensitive transcriptional route. In high-content analyses of breast tumor and non-tumor epithelial cell lines, cell–cell contact and cell-shape features, including cell and nuclear area and protrusiveness, were associated with nuclear localization of the NF-κB subunit p65/RelA. Perturbation experiments further identified RhoA-ROCK-myosin II signaling as critical for shape-dependent differences in NF-κB localization and oscillatory dynamics following TNFα stimulation [36]. Taken together, these studies support the involvement of multiple transcriptional regulators in breast cancer mechanobiology, including YAP/TAZ, MRTF/SRF, NF-κB, and β-catenin. Their responses depend on experimental context, with stiffness-dependent crosstalk demonstrated between YAP and β-catenin.

Mechanotransduction also intersects with receptor systems that are not necessarily primary force sensors. In HER2/Neu-initiated mammary epithelium, collagen crosslinking and matrix stiffening enhanced integrin clustering, focal adhesions, PI3K activity, and growth factor signaling, illustrating how mechanical context can amplify receptor tyrosine kinase-driven tumor progression [72]. Wnt/Frizzled and G-protein-coupled receptor pathways likewise converge on Hippo signaling. Alternative Wnt-FZD/ROR-Gα12/13-Rho signaling activates YAP/TAZ independently of canonical Wnt/β-catenin signaling [73], whereas LPA- and S1P-responsive G12/13-coupled receptors activate YAP/TAZ, and Gs-coupled agonists, such as epinephrine, inhibit YAP through LATS1/2 [74]. Accordingly, junctional β-catenin within cadherin complexes should be distinguished from nuclear β-catenin acting with TCF/LEF, and these receptor pathways are best viewed as mechanically modulated signaling networks rather than direct force sensors.

The combined effect of altered adhesion dynamics, cytoskeletal remodeling, and downstream mechanosensitive signaling enables breast cancer cells to maintain proliferative and invasive phenotypes under mechanical conditions that would otherwise restrain normal cells. This altered mechanosensitivity drives durotaxis, where cancer cells preferentially migrate up stiffness gradients, a critical mechanism facilitating metastasis from the primary tumor into the rigid stromal interface [75]. More recently, advancements by Al-Hilal et al. illustrate more explicitly the role of durotaxis in cancer proliferation, revealing instances of FAK-paxillin-mediated durotaxis through YAP signaling pathways as promotors of metastasis [76].

In addition to active sensing machinery, breast cancer cells also actively remodel their biomechanical environment, further altering their mechanosensing behaviour. Tumours and the surrounding stroma often develop increased tissue stiffness, heightened interstitial fluid pressure, and elevated solid stress, all of which feed back into aberrant mechanotransductive responses [6]. These changes often lead cancer cells to adopt a more aggressive phenotype. For example, cells migrating across collagen networks of differing pore sizes show increased contractility, dysregulated nuclear mechanotransduction via emerin translocation, and enhanced activation of YAP in cancer models [77]. Thus, normal and malignant mammary cells share many mechanosensory components, but cancer-associated mechanotransduction alters the contextual gating, duration, and downstream consequences of these signals, promoting self-reinforcing feedback among ECM remodeling, cytoskeletal tension, and proliferative or invasive cell states.

**Fig. 2:**
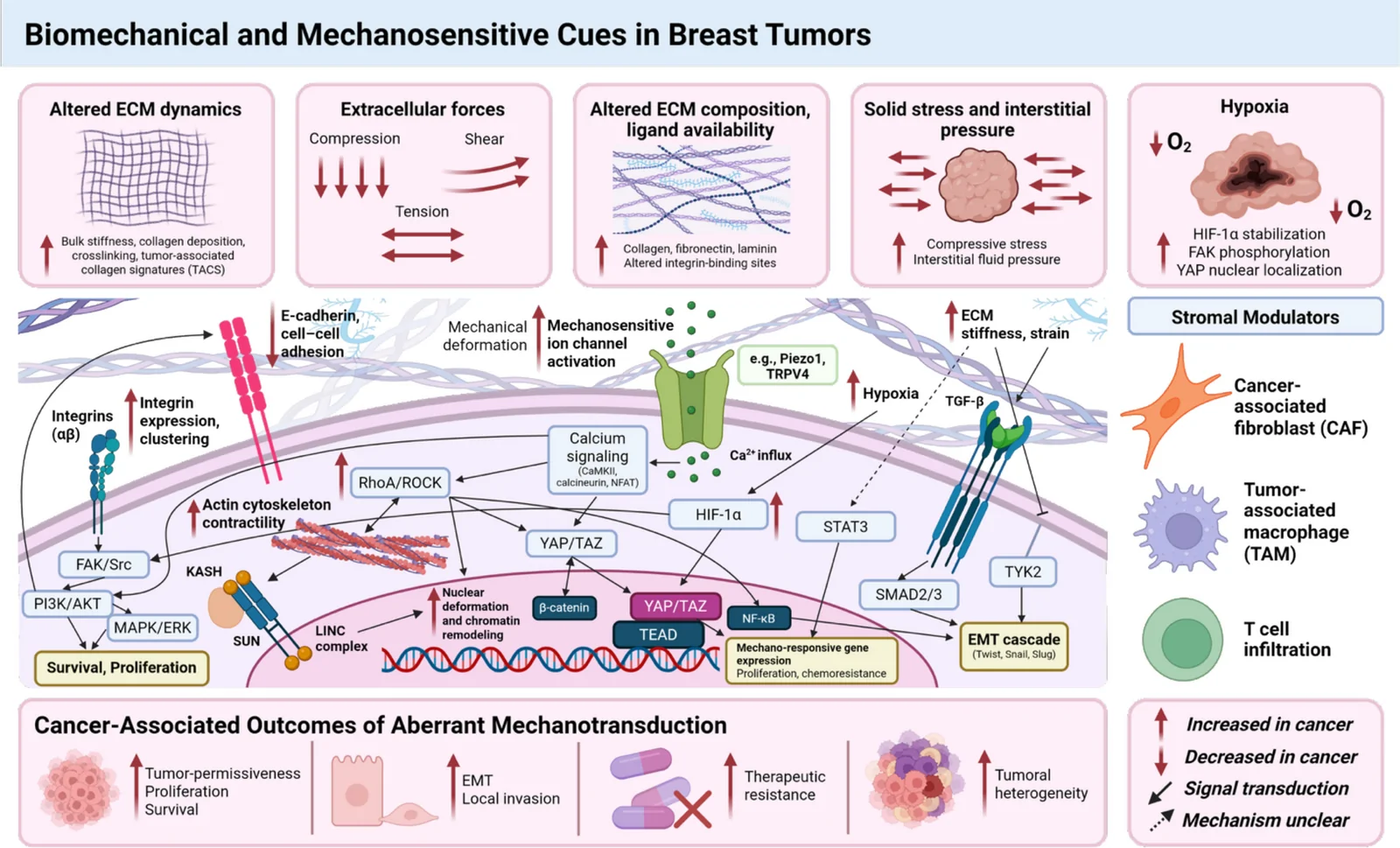
Context-dependent mechanotransduction factors in breast cancer. Variations in canonical mechanotransduction can induce tumorigenic phenotypes through multiple pathways. Increased ECM stiffness and remodeling, compression, tension, fluid shear, solid stress, interstitial pressure, hypoxia, and stromal or immune cell inputs all contribute to mechanosensitive signaling. Representative pathways include integrin–FAK/Src signaling, RhoA/ROCK-dependent actomyosin contractility, Piezo1- and TRPV4-mediated calcium signaling, altered cadherin and TGF-β signaling, and LINC-mediated transmission of cytoskeletal forces to the nucleus. These signals regulate mechanoresponsive transcriptional and EMT-associated programs, including YAP/TAZ activity, ultimately contributing to proliferation, invasion, metastasis, and therapeutic resistance. Critically, this figure shows abbreviated pathways, with context-dependent behaviour more robustly detailed in the body of the text. Figure created with BioRender

Viewed against the homeostatic framework outlined in Section I.A, Fig. 2 depicts a cancer-associated state in which ECM stiffening and reorganization, altered ligand presentation and cell-cell adhesion, solid and fluid stresses, hypoxia, stromal remodeling, and sustained mechanoresponsive transcription can amplify or prolong oncogenic signaling. These mechanisms represent context-dependent differences in signaling thresholds, response duration, and feedback to the ECM rather than binary distinctions between all normal and malignant cells, and they define features that more accurate preclinical models should be designed to reproduce.

### Mechanotransduction and therapeutic resistance

Differences between homeostatic and malignant mechanotransduction may have important consequences for therapeutic response. As discussed, in normal mammary tissue, mechanical signaling contributes to epithelial organization, differentiation, and tissue homeostasis, whereas the stiffened and remodeled ECM characteristic of breast tumors can sustain signaling programs associated with proliferation, survival, and invasion. Therapeutic response may therefore be influenced not only by the molecular characteristics of a tumor, but also by the mechanical context in which those molecular pathways operate. This consideration is particularly relevant to preclinical testing, as conventional culture systems frequently simplify or omit features such as tissue stiffness, physical confinement, and ECM architecture that influence mechanosensitive signaling *in vivo*.

Evidence for this relationship has been reported in the context of human epidermal growth factor receptor 2 (HER2)-targeted therapy. Lin et al. found that increasing ECM rigidity reduced the antiproliferative response of HER2-amplified breast cancer cells to the HER2/EGFR inhibitor lapatinib [78]. Increased matrix stiffness was accompanied by greater nuclear localization and transcriptional activity of YAP and TAZ, whereas depletion of either regulator increased lapatinib sensitivity. In an orthotopic model, YAP knockdown reduced tumor growth, while its combination with lapatinib showed a trend toward additional inhibition of growth over lapatinib alone, though this treatment did not reach statistical significance [78]. These findings suggest that inhibition of an oncogenic receptor pathway may be insufficient to fully suppress proliferative signaling when mechanically responsive pathways remain active. In this setting, stiffness-dependent YAP/TAZ signaling represents a potential parallel mechanism through which tumor cells may maintain growth despite HER2 inhibition.

Matrix mechanics may also influence responsiveness to conventional chemotherapy. Using 3D alginate hydrogels with tunable stiffness, Joyce et al. reported that increased matrix stiffness was associated with greater doxorubicin resistance in a TNBC cell line, MDA-MB-231, as well as increased nuclear localization of YAP [79]. Notably, the same stiffness-dependent response was not observed under conventional 2D culture conditions, suggesting that this drug-resistant phenotype depended on the interaction between tumor cells and their mechanical environment, rather than solely on an intrinsic resistance program [79]. This finding directly illustrates a limitation of conventional preclinical drug screening: a therapy may appear effective in a mechanically uniform culture system while failing to reproduce resistance mechanisms that emerge when tumor cells interact with a physiologically relevant ECM.

Mechanical regulation of therapeutic responses further involves signaling upstream of YAP/TAZ. Qin et al. demonstrated that matrix stiffness altered doxorubicin sensitivity in breast cancer cells through an integrin-linked kinase (ILK)–YAP signaling axis. Therein, increased stiffness promoted ILK-dependent YAP activation and was associated with a drug-resistant phenotype [80], further implicating that mechanically mediated resistance may arise through interconnected adhesion, cytoskeletal, and transcriptional pathways rather than through YAP/TAZ activity in isolation. This pathway redundancy presents an important challenge for conventional inhibition strategies: targeting a single signaling node may leave alternative mechanically regulated survival pathways intact.

Therapeutically, Murad et al. reported that focused, ultrasound-mediated mechanical disruption increased estrogen receptor-α expression and restored sensitivity to tamoxifen in tamoxifen-resistant, estrogen receptor (ER)+ breast cancer models, reducing tumor cell viability and proliferation in patient-derived xenografts [81]. Although the relative contributions of specific mechanotransductive pathways to this response remain unresolved, these findings support the broader concept that alteration of the physical tumor microenvironment can influence therapeutic sensitivity.

Collectively, these studies suggest that therapeutic efficacy in breast cancer may be shaped in part by the mechanical state of the tumor microenvironment, as well as by canonical oncogenic signaling. Increased ECM stiffness and associated activation of pathways involving YAP/TAZ, ILK, and cytoskeletal signaling provide alternative routes for maintaining tumor cell survival during pharmacologic inhibition. Conversely, modifying the mechanical environment may alter sensitivity to otherwise ineffective therapies. These observations expose a critical limitation of traditional preclinical models: rigid 2D substrates and mechanically uniform cultures can fail to reproduce the biomechanical conditions that generate therapeutic resistance, potentially leading to overestimation of treatment efficacy. Preclinical models incorporating physiologically relevant stiffness, stress relaxation, compression, ECM architecture, and stromal interactions are therefore necessary to identify mechanically mediated resistance mechanisms and to determine whether therapeutic strategies remain effective within the heterogeneous mechanical environment of breast tumors.

### Influence of the extracellular matrix and stromal factors

In breast cancer, the malignant microenvironment is often characterized by alterations in physical parameters, such as increased ECM stiffness, altered interstitial pressure, and solid stress [6]. These mechanical changes are not mere bystanders; rather, they actively reshape epithelial and stromal cell behaviour through reciprocal mechanosensing machinery. As such, understanding how the ECM mediates mechanotransduction is critical for modeling breast cancer in preclinical systems and for interpreting the functional consequences of tumour mechanics.

A key facet of the influence of the ECM lies in its biophysical properties: stiffness, fiber orientation, porosity, viscoelasticity, hydraulic resistance, and crosslinking, each of which affects how cells perceive and respond to mechanical cues [82, 83]. However, distinct from pure stiffness, the viscoelastic nature of the ECM, specifically its rate of stress relaxation, allows precancerous cells to mechanically remodel and invade the matrix, a property often lost in static, purely elastic culture models [84, 85]. Stiffening of the ECM in breast tissue is associated with increased deposition and cross-linking of collagen, fibronectin, and other matrix components, leading to elevated elastic moduli compared to normal mammary stroma [86]. In this stiffened state, breast epithelial or tumor cells exert greater traction forces through integrin-focal-adhesion-cytoskeleton linkages, activating downstream pathways such as Rho/ROCK, FAK, and YAP/TAZ [8]. Moreover, Pickett et al. recently revealed that fiber alignment and architecture, including collagen linearization, provide anisotropic mechanical cues that promote metastasis through shifting bioenergetics toward glycolysis [87]. These ECM properties, therefore, not only provide a scaffold, but act as active modulators of mechanotransductive signaling in breast cancer. However, collagen dynamics and bulk stiffness should not be conflated when considering the role of the ECM in breast cancer progression. For instance, crosslinking can increase matrix stiffness and integrin/PI3K signaling [72], whereas fiber alignment can direct protrusion orientation and predict 3D migration even when bulk stiffness is not the dominant factor [88].

Mechanosensing and mechanotransduction in breast cancer cells and their stromal companions are intimately linked to the ECM context and have direct implications for preclinical modeling. For example, *in vitro* culture systems that use soft hydrogels or stiff plastics may fail to mimic the physiological environment of a progressing tumor, thereby misrepresenting mechanotransductive signaling [86]. Similarly, 3D culture models that neglect fiber architecture, cross-linking, or matrix stress may underestimate the influence of ECM mechanics on cell phenotype. In breast cancer specifically, the increased ECM stiffness correlates with more invasive phenotypes and aberrant acinar morphogenesis in mammary epithelial models [6]. From a stromal perspective, cancer-associated fibroblasts (CAFs) respond to ECM mechanics through integrin signaling, leading to their dedifferentiation, aberrant remodeling of the ECM, and amplification of mechanotransductive feedback loops that induce cellular plasticity in epithelial cells [89]. Beyond the solid tumor itself, ECM dynamics have also been shown to control the metastatic cascade. Hu et al. characterized a form of stiffness-induced metastasis enabled by a JAK family kinase, TYK2. Stiff mechanical cues specifically induced TYK2 dissociation from the cell membrane into the cytoplasm, initiating a failure cascade that leads to the nuclear localization of TWIST1, ultimately promoting EMT and an invasive phenotype [90]. Thus, preclinical models must encompass not only biochemical cues, but also realistic biomechanical contexts like matrix stiffness, viscoelasticity, and fiber geometry, in order to faithfully mimic breast tumor mechanobiology and mechanosensitive metastatic cascades.

Beyond bulk and architectural properties, the identity, density, and presentation of cell-adhesive ligands constitute independent determinants of mechanotransduction. Ligand availability is not equivalent to matrix stiffness or crosslink density, however. Matrices with comparable elastic moduli may present different numbers or types of integrin-binding sites and thereby support different levels of integrin clustering, focal-adhesion assembly, cytoskeletal tension, and YAP localization. In fibronectin-coated polyacrylamide hydrogels, stiffness-dependent YAP translocation in a stem cell model occurred predominantly at intermediate ligand density, whereas low or high ligand densities governed YAP localization independently of stiffness through integrin- and cytoskeletal-tension-dependent mechanisms [91]. This is further supported in primary mammary stromal fibroblasts, where force transmission and nuclear YAP responses also differed between collagen I and fibronectin due to ligand-specific integrin-binding dynamics [92]. Breast cancer models intended to interrogate integrin/FAK or YAP signaling would therefore benefit from reporting ligand identity and density independently of bulk matrix modulus.

Toward such improvements, tumor-associated collagen signatures (TACS) provide a clinically relevant framework for describing the spatial organization of fibrillar collagen around breast tumours. The canonical framework progresses from increased collagen near emergent tumor foci (TACS1), to fibers aligned predominantly parallel to the tumor boundary (TACS2), and to locally perpendicular or radial fibers associated with invasion into the surrounding stroma (TACS3) [93]. Multiphoton imaging has further extended this framework to five larger-scale patterns (TACS4–8) at the invasive front. In a retrospective study of nearly 1,000 patients across training, internal validation, and external validation cohorts, a composite TACS1–8 score stratified disease-free survival [93]. These signatures may therefore provide critical structural benchmarks for evaluating whether preclinical models reproduce clinically observed collagen topology, although prospective validation would be required before clinical application.

Often facilitating such dynamics, several stromal cell populations contribute to the formation and remodeling of these ECM states. In mammary models, fibroblast-specific PTEN loss increased fibroblast activation and contractility, ECM deposition, and collagen alignment, whereas inhibition of contractility hindered the enhanced alignment phenotype [94]. Further, obesity-associated adipose stromal cells promoted collective breast tumor cell invasion through direct cellular contact, local collagen contraction, and matrix metalloproteinase (MMP)-dependent proteolytic remodeling [95]. Tumor-associated macrophages can also participate directly in matrix modulation: a stiff, fibrotic breast tumor microenvironment induced a TGF-β-directed collagen biosynthesis program in macrophages, with metabolic consequences that impaired CD8 + T cell function [96]. Further, Pascual et al. mapped mammary fibroblast states across normal development and breast tumorigenesis, identifying stage-specific ECM-remodeling populations, including Mgp+/Lox + fibroblasts predicted to promote collagen cross-linking and matrix stiffening [97]. Ultimately, the authors demonstrated that breast tumors adopt heterogeneous CAF states as the tumor advances, including ECM-producing and senescent myofibroblastic CAFs. Taken together, the cellular sources and regulators of the mechanical microenvironment change dynamically during tumor progression; fibroblasts, CAFs, adipose stromal cells, and macrophages can regulate aspects of ECM deposition, alignment, contraction, proteolysis, and fibrosis that directly influence cancer progression and outcomes.

Stromal remodeling and tumor growth also generate tensile strains that are mechanically distinct from stiffness, compression, and fluid shear. Spatial mapping of tumor solid stress has shown that growing tumors contain tensile and compressive regions, and that mechanical confinement by surrounding tissue contributes substantially to stress accumulation [98]. Fibroblast-mediated traction can further tension and align matrices rich in collagen and fibronectin, as illustrated by the contractility-dependent alignment exhibited in mammary fibroblast models [94] In addition, a 3D magnetic actuation platform applied cyclic tensile strain at controlled amplitudes and frequencies to breast cancer cells embedded in a fibrillar fibronectin matrix while evaluating YAP activity, cell morphology, metabolic activity, and matrix degradation. Following actuation, overall rates of ECM degradation in breast cancer cell line models reduced in response to the cyclic stretching forces [99]. Thus, models incorporating tensile loading would provide the most thorough context by reporting strain magnitude, frequency, and duration, rather than inferring tensile exposure from a bulk matrix modulus alone.

Likewise, hypoxia would most accurately be considered together with ECM mechanics rather than as an independent microenvironmental variable. In breast cancer models, stiff and hypoxic conditions acted cooperatively to promote stem-like marker expression and behaviour through ILK and PI3K/AKT signaling, with ILK depletion attenuating these responses [100]. A complementary study found that HIF-1α interacted with the integrin co-activator Kindlin-2, ultimately modulating P4HA1 and phosphorylated FAK expression in breast cancer cells, linking hypoxic signaling to collagen biogenesis and integrin-associated mechanics [101]. Additionally, HIF-1α has been demonstrated to promote nuclear translocation of YAP, further affecting mechanoresponsive signaling and changes to gene expression [102]. Models intended to examine hypoxia-mechanotransduction crosstalk should therefore vary oxygen tension and matrix mechanics jointly, and assess HIF-1α, ILK, integrin-FAK activity, and ECM remodelling as interconnected readouts.

Breast tissue is also heterogeneous at the organ scale, and mammographic density provides an imaging-based measure of the relative proportions of fibroglandular and fatty tissue. The 2025 edition of the Breast Imaging Reporting and Data System (BI-RADS) classifies breast composition as either: almost entirely fatty, containing scattered areas of fibroglandular density, heterogeneously dense, or extremely dense tissue [103]. However, mammographic density should not be equated directly with local ECM stiffness, although it is relevant to the tissue context in which mechanical signaling is studied. In a screening cohort of 37,839 women, African American women had higher adjusted odds of extremely dense breast tissue, but lower adjusted odds of heterogeneously dense breast tissue than non-Hispanic women of European ancestry [19]. However, a separate analysis by the Breast Cancer Surveillance Consortium found that the prevalence and population-attributable contributions of breast density varied by race and menopausal status [20]. These observations support consideration of density-associated tissue states in model design while underscoring that mammographic density alone does not explain population-level disparities.

Patient-associated variation also extends beyond stiffness and mammographic density to ECM and immune features. A 2025 bioRxiv preprint reported enrichment of collagen deposition and prognostically unfavourable T-cell signatures in hormone receptor-positive tumors from Black women. However, the T-cell signature was more strongly aligned with poverty burden, and the collagen findings represent measured deposition patterns rather than direct measurements of tissue mechanics [104]. These insights reinforce the need to distinguish self-identified race, genetic ancestry, and socioeconomic exposures, and to avoid treating group-level associations as universal or as proof of a causal mechanical mechanism.

## Preclinical modeling of breast cancer mechanotransduction

### Limitations of 2D cell culture

2D monoculture models are insufficient proxies for assessing breast cancer mechanotransduction, primarily due to the artificial introduction of stark variations and distortions in compliance, stiffness, geometry, and other absent or misrepresented mechanical forces that are exhibited *in vivo*. Fundamentally, culturing cells in a monolayer on GPa-stiff plastic creates an environment with mechanical forces that are often orders of magnitude beyond those in mammary tissue. This arbitrary constant can subsequently mask the true mechanosensitive signals and phenotypes demonstrated to contribute to tumorigenesis, tumor permissiveness, plasticity, and metastasis *in vivo*. This is particularly evident when considering that native mammary tissue exists in the kilopascal range, typically 1–10 kPa for healthy stroma versus over 15 kPa for the most stiffened tumors [4]. Beyond mere stiffness, 2D systems cannot simulate the compressive loads or steric hindrance provided by a 3D extracellular matrix. Without this physical confinement, downstream signaling, particularly the Rho–ROCK–YAP axis, is often pre-activated by the rigid substrate itself, interrupting critical mechanosensitive pathways that underlie tumorigenesis and metastasis [84, 105]. This baseline activation makes it nearly impossible to isolate the true functional effects of tumor-specific mechanical cues in a 2D monolayer culture setting (Table 1).

**Table 1:** Advantages and limitations of applying preclinical breast cancer models in mechanotransductive contexts

Preclinical model	Key advantages	Persistent limitations
2D monolayer culture	High throughput; standardized mechanical conditions	Low physiological relevance; exceptional relative stiffness (GPa); lacking heterogeneity and metastatic context
3D spheroids, organoids, and co-culture	Tunable matrix architecture; 3D confinement and remodeling	Technical variability; may oversimplify ECM and stroma; lacks circulation and organ colonization
Bioreactors and organs-on-chips	Dynamic cues controlled (shear, flow, compression, and cyclic strain); modularity enables metastatic modeling	Simplified relative to endogenous organs; precise forces and associated biological responses require validation
Bioprinting	Precise spatial control of mechanics, architecture, and cell distribution; highly reproducible	Bioink material constraints; tradeoffs among printability, shape fidelity, cell compatibility, and ECM fidelity
*In vivo* models	Near-native ECM, stroma, and vasculature; endogenous stresses and remodeling; natural force gradients and metastatic progression	Limited variable control; species-specific mechanics and anatomy
Computational modeling	Multiscale integration of spatial, molecular, and mechanical data; potential for patient-specific model designs	Requires heavy, functional validation against empirical samples; lack of AI interpretability and decision-making

### 3D *in vitro* cultures

Providing a step beyond monolayer culture and toward greater biomechanical relevance, tunable scaffolds, heterogeneous co-cultures, spheroids, organoids, and cultured explants have more promise as representative preclinical models. These platforms critically enable exploratory investigation of ancestry-associated signaling. Organoids, stromal–epithelial co-cultures, and similar preclinical constructs could investigate the effects of ancestry-specific annotations and of patient-derived epithelial, stromal, and immune cell interactions to more accurately recapitulate known ancestry-linked variations. For instance, reciprocal pairing of epithelial and stromal cells from donors with different, genetically verified ancestries under otherwise standardized ECM composition and mechanics could help characterize variations central to driving observed differences in breast cancer emergence and progression between populations.

This approach is consistent with reports of ancestry-associated variability in breast cancer gene expression and immune infiltration between women of African and those of European ancestries [12]. These and other mechanosensitive factors are implicated contributors to the increased incidence of TNBC and overall breast cancer mortality among women of African ancestry, particularly in the United States [2, 14]. Further contributing to this connection, PZP stromal cells were found to be enriched within healthy breast stroma from donors of African ancestry, while concurrently promoting epithelial cell invasion through fibronectin deposition and epithelial AKT activation [13]. Ultimately, genetically-informed patient ancestry, self-reported race, and social and environmental exposures ought to be documented as related variables, but not necessarily interpreted as interchangeable variables.

Further examination of biomechanical influence and variations can be evaluated through tight control of the biomechanical properties of the scaffold. Engineered 3D alginate matrices crosslinked with rat tail collagen I by Sharma et al. exhibit highly tunable viscoelastic properties that can be used to model how the rate of relaxation and strain influence transcriptional regulators of mechanotransduction, such as SP1 [106]. In breast cancer, the ability of the ECM to dissipate energy through stress relaxation is a critical determinant of cell behavior. High stiffness, fast relaxation, and high collagen density all promoted invasive behaviors in both breast cancer cells and non-tumorigenic controls. In the same study, phosphorylated SP1, regulated by PI3K and ERK1/2 activity, showed increased binding at loci of interest and reduced patient survival [106]. In a complementary approach, Bakhshandeh et al. employed a similar breast cancer hydrogel model to control 3D cell adhesion, matrix degradability, and bulk stiffness. The authors thereby discerned that increased scaffold stiffness induced nuclear localization of FHL2, ultimately increasing p21 expression, paclitaxel resistance, and rates of dormancy [107].

Spheroid models of cancer cells yield a more robust *in vitro* model of the heterogeneous layers of a solid tumor than monolayer culture alone [108]. By centrifuging cell lines in ultra-low attachment plates, many tumorigenic cells, including the breast cancer line, MCF7, can form dense clusters with layers of variable expression [109]. While spheroid assembly may only be practical for less aggressive cancer cell lines, substantial progress has recently been made in the generation of TNBC spheroids from MDA-MB-468 by Mohamedahmed et al. and from MDA-MB-231 by Huang, Yu, and Tang [110, 111]. These models are best for controlled or isolated mechanotransduction studies on variables such as stiffness, force transmission, and signaling pathways, and the emergence of more accurate TNBC models for these investigations provides critical insight into the mechanosensitive capacity of this highly invasive subtype. However, physical limitations and the homogeneous nature of spheroids suggest that the interpretation of known tumorigenic variables, such as ECM stiffness, may not recapitulate *in vivo* behavior in all cases [112].

Alternatively, breast carcinoma organoids often provide greater fidelity to the original sample, especially for basal-like and metastatic cases; however, challenges persist in the establishment of organoid models of less invasive phenotypes [113]. Using organoids cultured in domes of either basement membrane extract type 2 or collagen I, collagen I fiber density and alignment were found to strongly influence breast cancer cell invasion through a YAP-dependent feed-forward loop within the organoids [114]. Through mouse and human models, YAP was found to promote downstream signaling that increases collagen I alignment and tension. This increase in tension promotes further activation of YAP in a positive feedback loop that enables basal-like breast cancer cells to increase their invasive phenotype in collagen I [114]. Organoid models are, thus, preferable *in vitro* models for physiologically relevant, heterogeneous interactions that influence mechanotransduction. However, significant heterogeneity exists not only within individual organoid models, but between breast cancer organoids. This aspect of organoid models reflects the heterogeneous nature of breast cancer, but may also introduce confounding variables that may be difficult to adjust for at scale [115].

Although engineered matrices can reproduce selected mechanical properties, epithelial-only systems remain limited in their ability to capture the reciprocal interactions between tumor cells and the stromal compartment. To reflect the heterogeneity of breast cancer tissues, 3D cultures have incorporated stromal–epithelial co-cultures or primary tumor explants. Co-culture models have demonstrated that stromal composition can directly alter epithelial organization and tumor-associated phenotypes, with fibroblasts, endothelial cells, and immune populations providing distinct signals that are not captured by epithelial monocultures alone [116]. In one such example, Silva Krause et al. developed a 3D model using MCF10A mammary epithelial cells from a patient of European ancestry, and co-cultured them with primary human mammary fibroblasts isolated from reduction mammoplasties in either type I collagen or a Matrigel–collagen matrix. After up to six weeks in culture, histological and ultrastructural analysis demonstrated the formation of ductal and alveolar structures. Notably, fibroblasts accelerated the initial formation of epithelial structures in collagen gels and were required for ductal structure formation in the Matrigel–collagen system, demonstrating that stromal cells can directly regulate epithelial morphogenesis rather than simply providing additional cellular complexity [117].

Primary explants provide an even closer representation of native tumor phenotypes by retaining heterogeneous cell populations and cell surface characteristics that can be lost when modeled in traditional culture. Donnenberg et al. profiled three metastatic breast cancer explants derived from pleural effusions and found that clonogenic explant cells expressed both mesenchymal-associated markers, including CD44, CD51/CD61, CD54, CD90, and CD105, and epithelial-associated markers, including EpCAM/CD326. The explant surface proteome was significantly correlated with normal mesenchymal stromal cells and transformed mammary epithelial cells, whereas MCF7, MDA-MB-231, and BT-474 cell lines showed no significant correlation with the explant phenotype [118]. Together, these studies underscore the importance of incorporating stromal and patient-derived cellular complexity into 3D breast cancer models to more faithfully reproduce tissue organization, heterogeneity, and tumor-associated phenotypes than epithelial monocultures alone.

Further, even models that incorporate stromal components may represent the ECM as a relatively static material, despite the capacity of tumor cells to actively modify its architecture and mechanical resistance during invasion. In addition to responding to ECM stiffness and fiber organization, breast cancer cells remodel the surrounding matrix through proteolytic degradation. MMPs, particularly membrane-type MMPs, such as MMP14/MT1-MMP, provide one mechanism by which tumor cells can locally reduce the physical resistance of the ECM during invasion. In a 3D collagen I model of mammary epithelial invasion, MMP14-mediated degradation locally reduced collagen microelasticity at invasive fronts, thereby decreasing the mechanical resistance encountered by invading cells [119]. These findings indicate that ECM mechanics are not simply external properties imposed on tumor cells, but can themselves be modified through localized proteolysis.

Consistent with this concept, recent breast cancer spheroid studies have shown that YAP activity, actomyosin contractility, and MMP activity collectively regulate collagen remodeling and invasion [112]. Pharmacological inhibition of MMPs reduced collagen fiber alignment and invasion in lower-stiffness collagen matrices, suggesting that proteolytic ECM remodeling acts in concert with cytoskeletal force generation to establish a matrix environment permissive to tumor-cell migration. This relationship is also stiffness-dependent: increased matrix stiffness activated ERK1/2 and YAP, but did not necessarily increase invasion, underscoring that mechanosensitive signaling alone may be insufficient to predict invasive behaviour without accounting for the capacity of cells to physically remodel the surrounding matrix [112]. MMP-dependent remodeling may therefore function as a feedback mechanism in which mechanotransduction promotes contractility and matrix remodeling, while proteolytic degradation subsequently alters local ECM architecture and mechanical resistance.

Single-cell and collective invasion should also be distinguished in 3D models. At the single-cell level, adhesion, contractility, actin dynamics, protrusive activity, and matrix remodeling combine in distinct coordination states that produce different relationships between migration speed and persistence [120]. In collective breast cancer invasion, K14-positive basal-like leader cells guide cohesive groups, indicating that coordinated interactions among heterogeneous epithelial subpopulations remain central to the invasive program [121]. Matrix fiber alignment can further direct protrusion orientation and alter the utility of MT1-MMP, linking traction-mediated and proteolytic remodeling to the mode of invasion [88].

Accordingly, incorporating MMP-sensitive or otherwise enzymatically degradable matrices into spheroid, organoid, and stromal co-culture models may provide a more physiologically representative framework for studying breast cancer invasion than static matrices in which ECM stiffness and architecture remain fixed. Nevertheless, increasing model complexity does not inherently ensure physiological relevance, and these systems should ultimately be evaluated against experimentally measured properties of human tumors.

Conversely, the effects of a pro-tumor biomechanical microenvironment transitioning to less tumor-permissive conditions have additionally emerged as a frontier of breast cancer therapeutics. Specifically, stromal normalizers, agents that restore stromal architecture more closely to healthy parameters, have also been integrated into advanced preclinical models to improve the measurement of mechanosensing. In 3D culture systems and organotypic models, stromal normalization allows investigators to tune mechanical parameters, such as elastic modulus, fiber alignment, and interstitial pressure, while maintaining physiologically relevant cell–matrix interactions. Most recently, Neophytou et al. combined the mechano-therapeutic compound, ketotifen, with ultrasound sonopermeation to modify the tumor microenvironment, enhancing the efficacy of Doxil and immune checkpoint inhibition in murine breast tumor models [122]. Studies using normalized fibroblast populations or ECM scaffolds derived from modulated stroma demonstrate more reproducible mechanotransductive responses, including altered nuclear mechanosignaling and chromatin organization in breast cancer cells [17]. These studies suggest that stromal normalization may offer an underexplored opportunity to investigate the sensitivity and interpretability of assays designed to quantify mechanoresponsive factors, such as force transmission, cytoskeletal remodeling, and gene expression.

Validation of 3D breast cancer models should therefore extend beyond confirmation of overall morphology to establish whether a model reproduces the mechanical, molecular, and functional characteristics of the tumor of origin. At the physical level, hydrogel and organoid systems should be benchmarked against patient tissue for parameters including elastic modulus, viscoelasticity, collagen density, fiber organization, and matrix composition. Atomic force microscopy has demonstrated measurable differences in the elastic properties of malignant and nonmalignant human breast tissue, providing an experimentally accessible mechanical benchmark for engineered matrices [123]. At the molecular level, validation should include mechanotransductive readouts such as YAP/TAZ localization, FAK/Src activation, Rho-ROCK signaling, cytoskeletal organization, and nuclear morphology. These readouts should also be interpreted within the appropriate dimensional context, as mechanotransduction observed on rigid 2D substrates is near-certainly misrepresentative of signaling in 3D tissue. Lee et al. furthered this validation need when they found that, in the mechanically-tunable 3D mammary cultures, stiffness-dependent responses occurred without corresponding YAP activation. Similarly, YAP activation was not observed in the ductal carcinoma *in situ* specimens studied; rather, additional experiments implicated STAT3 in the observed mechanotransduction in 3D culture [70]. Thus, 3D model validation should be performed within a relevant 3D context, and ideally compared with their parental tumors for histopathology, receptor expression, genomic features, cellular heterogeneity, and therapeutic response. Validation studies of similar models further support the greater concordance between 3D preclinical models and *in vivo* tumors. Breast cancer organoid biobanks have demonstrated sustained histological and genomic continuity with their parental tumors, together with drug response patterns that corresponded with xenograft and patient responses [115]. More recent patient-derived organoid studies have similarly reported preservation of parental histology, receptor status, genomic alterations, and clinical drug-response patterns [124]. Collectively, these findings support 3D model validation strategies across mechanical, molecular, architectural, and functional levels, rather than relying on morphology or viability alone.

### Bioreactors and organ-on-a-chip systems

Bioreactor systems can be engineered to yield controlled, dynamic mechanical environments that are uniquely suited to probing the mechanotransduction mechanisms underpinning breast cancer progression and response to therapy across scales. Unlike static cultures, bioreactors can apply physiologically relevant shear stress, interstitial flow, compression, and cyclic tensile strain to cancer cells embedded within 3D extracellular matrices, thereby modeling mechanical stressors present *in vivo* [24]. Specifically, breast cancer cells subjected to defined shear stress in a bioreactor system displayed increased proliferation, invasive morphology, chemoresistance, and upregulation of mechanotransductive genes, such as *PLAU*, compared with static controls, demonstrating how mechanical forces can drive malignant phenotypes through mechanosensitive pathways [24]. Similarly, compression bioreactors have shown that static compressive stress enhances expression of matrix-remodeling and adhesion genes, suggesting that mechanical loading can potentiate metastatic traits and alter cell–ECM interactions relevant to tumor progression [25].

Similarly, 3D organ-on-a-chip (“chip”) systems provide similarly dynamic, physiologically relevant platforms that replicate these biomechanical cues with high spatiotemporal precision. These engineered systems integrate microfluidics, biomaterials, and living cells within controlled architectures, enabling quantitative studies of how tumor cells sense and respond to mechanical forces that resemble those *in vivo* [125]. Recent advances in breast cancer chip models have illuminated the interplay between ECM stiffness and tumor cell behaviour, particularly EMT, invasion, and metastatic potential. Breast cancer chip platforms have been used to tune matrix rigidity and observe mechanosensitive signalling via integrin–FAK pathways, YAP/TAZ nuclear translocation, and downstream transcriptional reprogramming [126]. Microfluidic perfusion within these systems allows for the simulation of interstitial flow, mimicking lymphatic drainage, and shear stress, mimicking vascular flow, which modulate cancer cell alignment, migration, and drug response. Through chip models, Liu et al. were recently able to simulate organotropism of immortalized cell lines, providing support for the physiological relevance of chip models [127]. Incorporating similar stromal and immune components into micropatterned chips can further enhance the physiological relevance of mechanotransduction studies by more accurately reproducing mechanical signaling between tumor, fibroblast, and endothelial cell populations [128].

To further advance such models, immune cells ought to be incorporated as mechanosensitive participants whenever feasible, rather than solely as passive components of chip-based tumor models. Piezo1-dependent Ca^2+^ influx and actin remodeling contribute to macrophage stiffness sensing and polarization [129], whereas T cells cultured in 3D microporous alginate scaffolds with independently controlled microporosity exhibited greater activation, proliferation, and migration speed in stiffer matrices than in softer matrices [130]. Ideal chip systems could therefore vary stiffness, porosity, collagen architecture, and flow, while measuring macrophage activation, matrix deposition, T cell migration, and effector function. The interplay of these elements will be necessary to model and thereby capture reciprocal feedback between immune mechanosensing channels and tumor phenotypes.

These dynamic, reproducible developments yield significant implications for preclinical breast cancer modeling. By reconstructing the dynamic mechanical microenvironment of breast tumors, bioreactors and chip systems bridge the gap between *in vitro* assays and complex animal models, improving the predictive power of mechanobiology-driven drug screening at scale. Such platforms enable the critical testing of anti-cancer agents that target mechanoresponsive pathways, such as inhibitors of FAK, YAP/TAZ, or matrix crosslinking enzymes, under conditions that mimic *in vivo* stiffness and fluid dynamics. Moreover, the modularity of chip systems facilitates integration with patient-derived cells, paving the way for personalized medicine approaches that account for interpatient variability in tumor biomechanics. Collectively, these advances underscore the transformative potential of organ-on-a-chip models for elucidating mechanotransduction in breast cancer and refining preclinical evaluation of therapeutics targeting tumor mechanosensing.

The dynamic control afforded by bioreactors and chip systems makes these platforms particularly well suited to modeling mechanically regulated stages of the metastatic cascade. Multi-organ or tissue-specific chip systems may further incorporate endothelial, stromal, and mechanical components representative of common metastatic sites, including lung and bone, as demonstrated by Liu et al. [127]. Their bio-inspired chip model for assessing breast cancer organotropism among circulating tumor cells could similarly be adapted for assessing the impact of mechanobiological forces in the metastatic cascade. The authors specifically noted that the modularity of this system would allow for the implementation of tunable mechanical properties, thereby enabling investigation of how mechanical differences among primary and secondary tissue niches may influence extravasation and metastatic colonization [127]. Similarly, microfluidic breast cancer metastasis models, incorporating heterogeneous cell populations and scaffold parameters, have demonstrated that substrate stiffness can increase extravasation incidence and MMP9 secretion throughout the channels of the device [131].

The utility of bioreactor and chip models, however, depends on validation of both the applied mechanical stimulus and the resulting biological response. For bioreactor systems, experimental parameters such as shear stress, fluid velocity, pressure, strain, or matrix deformation should be compared with the intended conditions rather than inferred solely from device geometry or pump settings. Novak et al., for example, characterized a 3D breast cancer bioreactor using finite-element modeling and applied a continuous shear stress of 5.4 dynes/cm^2^ to hydrogel-encapsulated breast cancer cells for 72 h. The authors validated their experimental parameters computationally and validated their results across cell lines of different patient ancestries. The authors found that this mechanical stimulus increased proliferation, invasive morphology, paclitaxel resistance, and PLAU expression, bolstered by the concurrent mechanical, molecular, and functional readouts and analyses [24].

Validation of bioreactor and chip-based models may therefore be considered across three complementary levels: engineering fidelity, referring to whether the device reproduces the intended force, flow, or deformation field; molecular fidelity, referring to whether relevant mechanotransductive pathways are engaged appropriately; and functional fidelity, referring to whether the system reproduces biologically relevant outcomes such as invasion, extravasation, proliferation, or therapeutic response. For models intended to study metastasis, functional validation is particularly important because successful reproduction of a mechanical stimulus does not necessarily establish that the corresponding metastatic behavior is physiologically representative. Where feasible, these mechanical and biological outputs should be benchmarked against patient-derived tissues or clinically measured mechanical parameters rather than against another engineered model alone.

### Bioprinting

Bioprinting has emerged as a powerful platform for interrogating mechanotransduction in breast cancer by enabling precise spatial control over the physical properties of the tumor microenvironment in three dimensions. Unlike self-organizing organoids, bioprinting allows researchers to engineer precisely defined, highly replicable architectures. By tuning exact parameters for stiffness, matrix composition, and fiber alignment, bioprinting can reliably recapitulate key biomechanical features of tumor progression at the mesoscopic scale [132, 133]. This level of control is particularly advantageous for studying how breast cancer cells sense and respond to mechanical heterogeneity, including detailed gradients in ECM stiffness that are known to drive malignant behavior [55, 134].

Bioprinted breast tumor models can also reproduce spatial heterogeneity in ECM mechanics. Yuan et al. observed relatively softer cancer cell-rich regions and stiffer, collagen-rich surrounding stroma in a spatially patterned, bioprinted model [135]. Similarly engineered gradients provide a robust platform to study durotaxis and its role in breast cancer invasion and metastasis [136]. When collagen-rich or other fibrillar bioinks are used with appropriate printing strategies, fiber organization can be introduced to model anisotropic ECM architectures that influence integrin signaling, cytoskeletal tension, and downstream mechanotransduction pathways such as YAP/TAZ [137].

However, these opportunities are currently constrained by bioink chemistry and rheology. In extrusion-based bioprinting, suitable flow behaviour and stabilization after deposition are needed to preserve filament geometry [138, 139]. Higher polymer concentrations or more extensive gelation before extrusion can increase shear stress and reduce cell viability, depending on the formulation and printing conditions [139]. Alginate specifically illustrates some of these design constraints. Its mild ionic gelation and tunable mechanics are useful for construct fabrication, but unmodified alginate lacks intrinsic mammalian cell-adhesive ligands [140] and is resistant to mammalian enzymatic degradation [141]. Further, its polymer network also does not inherently reproduce the fibrillar architecture of native collagen. RGD functionalization can introduce cell-adhesion sites [140], while partial oxidation can promote hydrolytic degradation and alter gel mechanics [141]. The alginate–collagen I matrices discussed in Section II.B provide a complementary material design: they incorporate a fibrillar collagen network while allowing independent tuning of stiffness and stress relaxation through alginate crosslinking and molecular weight, respectively [106]. Translating such material designs into bioinks requires formulation-specific evaluation of rheology, shape fidelity, cell viability, and the intended biological functions [139].

Rigorous validation is particularly important for bioprinted models, as geometric reproducibility alone does not necessitate biological fidelity. Printed constructs should therefore ideally be characterized for spatial stiffness, ECM composition, fiber architecture, porosity, cellular distribution, and vascular organization, with these features compared, where possible, with corresponding properties of human breast tumours. The spatially-defined, bioprinted breast tumor model developed by Yuan et al. comprised MDA-MB-231 cells, endothelial cells, and mammary CAFs. From this engineered co-culture model, they demonstrated spatial differences in ECM stiffness, angiogenesis, and ECM remodelling signatures that reproduced empirical features of a heterogeneous tumor microenvironment [135]. These spatial differences were also associated with heterogeneous chemotherapy responses, providing a functional benchmark in addition to architectural and mechanical characterization. Similarly, Desigaux et al. found that incorporation of CAFs into a bioprinted breast cancer model conferred anticipated increases in ECM remodelling and stiffness, and further enhanced the radioresistance of cancer epithelial cells in the system [142]. Together, these studies indicate that validation of bioprinted models ought to extend beyond printed architecture and mechanics to include multiple core benchmarks validating the establishment of anticipated microenvironmental niches that enable stromal remodeling, mechanotransductive signaling, and varied therapeutic responses. Accordingly, a construct that reproduces tumor geometry but does not recapitulate key features of ECM remodelling or treatment response may be considered structurally representative, but biologically incomplete.

### *In vivo* models

Preclinical animal models have long been used to study breast cancer mechanobiology in a more physiologically relevant environment. However, important limitations remain when translating findings from animal studies to human disease. Murine models predominate because of their amenability to genetic manipulation and rapid breeding, yet their small size, distinct tissue mechanics, and differences in mammary anatomy produce mechanical environments that differ substantially from those of human breast tissue. Most notably, differences in tumor–stromal interactions and ECM composition can confound the translation of some findings to clinical contexts [143]. Nevertheless, murine models offer an important advantage in that mechanical forces can be manipulated and quantified within an intact tumor microenvironment.

Recently, Boyle et al. subjected early-stage MMTV-PyMT mammary tumor fragments to 20 kPa compressive stress for 30 min before orthotopic transplantation into the mammary fat pads of congenic mice. After 4 weeks, compressed tumors exhibited greater tumor area and burden, with compression activating the mechanosensitive ion channel Piezo1 and downstream Rho–ROCK signaling [58]. Piezo1 silencing abolished compression-induced Rho–ROCK activation and the associated tumor-promoting effects, while transient compression produced persistent changes in histone acetylation. These findings suggest that mechanical loading may establish an epigenetic form of mechanical memory that might not have been captured in a less comprehensive model [58]. Complementing these compression experiments, Zhang et al. developed an intravital approach to quantify endogenous solid stress in orthotopic MCa-M3C and E0771 breast tumors using fluorescent, deformable polyacrylamide beads as stress sensors [144]. Solid stress varied substantially with spatial scale, with tissue-scale principal stresses approximately 8.5-fold higher in MCa-M3C tumors and 5.3-fold higher in E0771 tumors than those measured at the cellular scale alone. Thus, by deploying these biosensors in an orthotopic model, the authors determined that local tissue force transmission strongly influences the mechanical forces experienced by individual tumor cells [144].

These findings briefly illustrate the capacity of *in vivo* murine models to capture both experimentally imposed and endogenous mechanical stresses while preserving the stromal, vascular, and tissue architecture that governs force transmission and mechanosensitive signaling. In particular, the ability to follow tumor progression from the primary mammary site to distant organs enables some of the most comprehensive investigations of how mechanical conditions drive metastatic dissemination and organotropism [144].

Alternatively, porcine models offer a more compelling alternative for mechanosensitive preclinical modeling due to their anatomical and physiological similarity to that of humans, offering increased translatability between species [145]. Pigs share greater similarity with humans in overall tissue size, organ structure, mammary architecture, and genomic orthology than rodents, which can provide more clinically relevant breast tissue models, stromal–tumor mechanical interactions, and ECM composition [146]. Among other similarities, porcine mammary tissue shares with humans the presence of terminal ductal lobular units dispersed throughout fibrous tissue [147]; murine models feature a less complex ductal network and a more adipose-rich mammary pad [146]. Crucially for breast cancer modeling fidelity, the transcriptome of developing mammary tissue in post-natal pigs mirrors known oncogenic expression changes and markers in human breast cancer [148]. These structural similarities more accurately reflect biomechanical factors of interest in human breast cancer: solid stress distributions, matrix composition, and average Young’s modulus of breast tissue, in a context that is not similarly feasible in mice [149].

However, species-specific limitations remain. Yucatan porcine mammary tissue exhibits many fewer milk-producing acini in the lobules and less well-defined ligamentous architecture than humans [150]. Ultimately, the factors of mechanical and structural similarity are critically important when investigating the biomechanical effects of pathology and intervention, including the substantially lower tumor-to-body-mass ratio in porcine models compared to murine models [151]. However, the higher cost, timeline, and resource burden of maintaining porcine models is often infeasible for early preclinical studies, particularly when compared to murine models [151].

Thus, validation of small and large animal models of breast cancer mechanotransduction should focus on whether the magnitude, spatial distribution, and cellular consequences of mechanical forces are representative of those observed in human tumors. Bulk tumor stiffness alone is insufficient for this purpose. Accordingly, animal models should be benchmarked against human measurements of elastic modulus, collagen architecture, solid stress, interstitial pressure, and tumor deformation, together with molecular readouts of mechanotransduction such as YAP/TAZ activity, FAK/Src signaling, Rho–ROCK activation, Piezo1 and TRPV4 signaling, and nuclear deformation to most accurately reflect the biological question under investigation.

Complementing these approaches, *ex vivo* precision-cut tumor slice cultures and *in vivo* patient-derived xenograft models can provide additional platforms for investigating breast tumor architecture and interactions with the surrounding microenvironment in a context similar to that of the original specimen. Briefly, microdissection sampling of breast tumors has recently been used by Sivakumar et al. to preserve ECM composition and stromal interactions, revealing distinct, time-sensitive stromal profiles of immune infiltration and activation across cancers [152]. Similarly, Hu et al. employed patient-derived xenograft models of TNBC in immunodeficient mice to discern the role of TYK2 in enabling the metastatic cascade from biomechanical signaling alone. The use of xenograft models was critical for preserving original tumor expression and structure, while experimentally assessing stiffness-dependent metastatic events and organotropism upon inhibition of TYK2 [90].

*Ex vivo* platforms therefore provide a valuable opportunity to study biomechanical niches in a physiologically relevant context, while retaining patient-specific heterogeneity, which is often lost in synthetic models [153]. These platforms bridge *in vitro* systems and *in vivo* patient tumors for investigating the mechanical regulation of breast cancer progression at a systems level. To that end, animal models and *ex vivo* samples provide a near-optimal source of heterogeneous mechanical, biochemical, and structural metrics for validating synthetic models of breast tumor mechanotransduction in the absence of specific patient data.

### Computational modeling and artificial intelligence

Recent advances in computational modeling and AI are providing rapidly accelerating avenues for capturing mechanotransduction dynamics in breast cancer by integrating gene expression at a systems-level and advancing our understanding of topographical and mechanical heterogeneity. However, the breadth of available and emerging models cannot be comprehensively reported within the scope of this review. Rather, a selection of models is presented, with an emphasis on exploring the breadth of relevant applications of emerging computational biology workflows, especially those which incorporate AI and machine learning approaches.

Beginning at the fundamental scale of gene expression, GEMDiff uses a diffusion-based workflow to simulate transitions between normal and tumor gene expression states in breast cancer, thereby enabling identification of core gene sets that may respond to mechanical cues or drive mechanobiological phenotypes [154]. Although GEMDiff is not explicitly engineered for mechanical microenvironment parameters, its ability to model perturbations between expression states offers a valuable platform to integrate mechanotransduction-related gene regulatory networks (e.g., YAP/TAZ, integrin–FAK, cytoskeletal genes) and simulate how mechanical signals might shift tumor cell transcriptomic states. By embedding mechanical sensor and effector modules into such workflows, computational frameworks like GEMDiff can help to predict how mechanical input might lead to downstream gene expression changes in breast cancer cells [154].

Complementing transcriptomic diffusion models, the STIFMap approach, developed by Stashko et al., offers a spatially resolved map of mechanical heterogeneity within human breast tumor tissue sections and links those data to mechanosensitive phenotypes [27]. STIFMap uses a convolutional neural network to predict matrix elasticity at micron resolution across tissue sections, registering local stiffness with biomarkers of EMT and other mechanosensitive processes. This spatial computational method provides a rich dataset of local stiffness heterogeneity that can be fed into mechanotransduction models, either agent-based, finite-element, or network-based, to simulate how discrete patches of elevated stiffness influence cell behaviour, signalling activation, and emergent invasion or drug resistance patterns [27].

Beyond data-driven AI approaches, physics-based modeling, specifically finite element analysis (FEA), remains indispensable for calculating the actual mechanical loads experienced by tumor cells. While STIFMap can predict material properties, such as the elastic modulus, FEA is required to translate these properties into stress and strain fields under physiological loads, including solid stress from tumor growth or interstitial fluid pressure. For instance, Wang and Kesavadas combined FEA with artificial neural networks to predict breast deformation and changes in tumor position associated with patient posture and tissue composition, providing a more mechanistic link that purely correlative AI models may miss [155].

However, data-driven approaches alone cannot establish how mechanical forces generate cellular deformation or tissue-scale stress. Mechanistic computational frameworks are therefore needed to connect predicted material properties with the physical forces experienced by individual cells and tissues. Agent-based and continuum models have been used to simulate collective cell migration while incorporating cell–cell interactions, matrix confinement, and environmental mechanical cues [156, 157]. Specifically, Collins et al. used a stochastic model of small cell clusters migrating through 3D fibrous matrices to demonstrate that optimal collective migration strategies depend jointly on extracellular conditions and the strength of intercellular mechanical coupling [156]. Similarly, Yeoman and Katira developed a stochastic algorithm for long-term migration path persistence that represents cell polarity and mechanosensitive activity together with matrix fiber density, stiffness, alignment, and binding-site density [157].

More detailed 3D models have further incorporated cell–matrix adhesion, nuclear deformation, steric confinement, and ECM degradation, demonstrating that cell migration cannot be assessed solely as a function of matrix stiffness. Merino-Casallo et al. integrated protrusion dynamics, cell–matrix adhesion, nuclear deformability, steric confinement, and ECM degradation in a mechanistic model of 3D migration, demonstrating that migratory behaviour depends on the combined cellular and matrix context rather than stiffness alone [158]. These approaches are particularly relevant to understanding the multivariable space of mechanotransduction-induced breast cancer invasion, in which collagen architecture, matrix porosity, cellular contractility, and proteolytic remodeling collectively determine the mechanical constraints encountered by tumor cells. At the tissue scale, newer 3D frameworks, such as SimuCell3D, enable computational representation of cell growth, proliferation, ECM, nuclei, and heterogeneous tissue mechanics, providing a foundation for multiscale analysis of tumor architecture and force transmission [159].

Computational models therefore require particularly careful validation, as agreement with a training dataset or selected experimental outcome does not in itself establish biological validity. Mechanical predictions should first be tested against independent measurements of tissue stiffness, collagen architecture, deformation, or solid stress obtained from human breast tumours. Atomic force microscopy measurements of human breast tissue provide one potential benchmark for local elastic properties [123]. Mechanical agreement alone, however, is insufficient; predicted mechanical states should also be compared with spatially matched molecular phenotypes, including YAP/TAZ localization, FAK activation, cytoskeletal organization, EMT markers, and mechanosensitive gene expression. Thus, such models should additionally undergo functional validation using independent samples and biologically or clinically relevant outcomes, such as invasion, metastatic behaviour, or therapeutic response. Computational models ought to be further evaluated on their ability to reproduce experimentally observed relationships among mechanical inputs, mechanotransductive signaling, and functional outcomes rather than on the accurate prediction of any single parameter in isolation.

## Conclusions and future perspectives

Mechanotransduction has emerged as a central axis of breast cancer biology, informing how mammary cells integrate mechanical and biochemical information during homeostasis and into cancer progression. The evidence reviewed here indicates that cancer-associated mechanotransduction does not arise from an entirely distinct set of sensors, but from context-dependent changes in the thresholds, duration, coupling, and consequences of shared pathways. Cell–ECM and cell–cell adhesions, the glycocalyx, mechanosensitive ion channels, Rho-family GTPases, the actin cytoskeleton, LINC-mediated nuclear force transmission, and transcriptional regulators, including YAP/TAZ, MRTF/SRF, NF-κB, and β-catenin, collectively shape epithelial organization and plasticity. In tumors, these pathways are further influenced by ECM stiffness and viscoelasticity, ligand availability, collagen organization, compression, tension, fluid flow, hypoxia, proteolytic remodeling, and stromal and immune populations. Their combined effects can promote persistent growth, invasion, metastatic dissemination, and mechanically mediated therapeutic resistance. Critically, ancestry-associated molecular, stromal, collagen, and architectural tissue differences often add further heterogeneity and accelerate proliferation, although their mechanistic contributions remain incompletely characterized, representing a critical gap in biological understanding and patient outcomes.

Addressing these challenges requires a shift toward more physiologically relevant preclinical models capable of capturing the complexity and heterogeneity of tumor biomechanics. However, no single preclinical platform can reproduce this multiscale environment in its entirety. 2D cultures offer high throughput, but distort stiffness, geometry, and force transmission. 3D matrices, spheroids, organoids, and co-cultures can model confinement, ECM remodeling, and tumor–stromal heterogeneity, while bioreactors and organ-on-a-chip systems introduce tunable flow, shear, compression, tensile strain, compartmentalization, and selected metastatic assessments. Bioprinting permits spatial patterning of mechanics and cell populations, subject to the chemical and physical limits of the bioink and bioprinter; *in vivo* models preserve more intact tissue architecture, but are hindered by species-specific variations; and computational approaches connect spatial measurements, signaling networks, and predicted stresses across scales, though empirical validation is required and explainer models have yet to render AI decision-making fully interpretable. Model selection should therefore be guided by the biological question and mechanical phenomenon to be reproduced rather than by platform complexity alone. Across model classes, physiological relevance requires validation of both the implemented mechanical conditions and the responses they elicit.

**Fig. 3:**
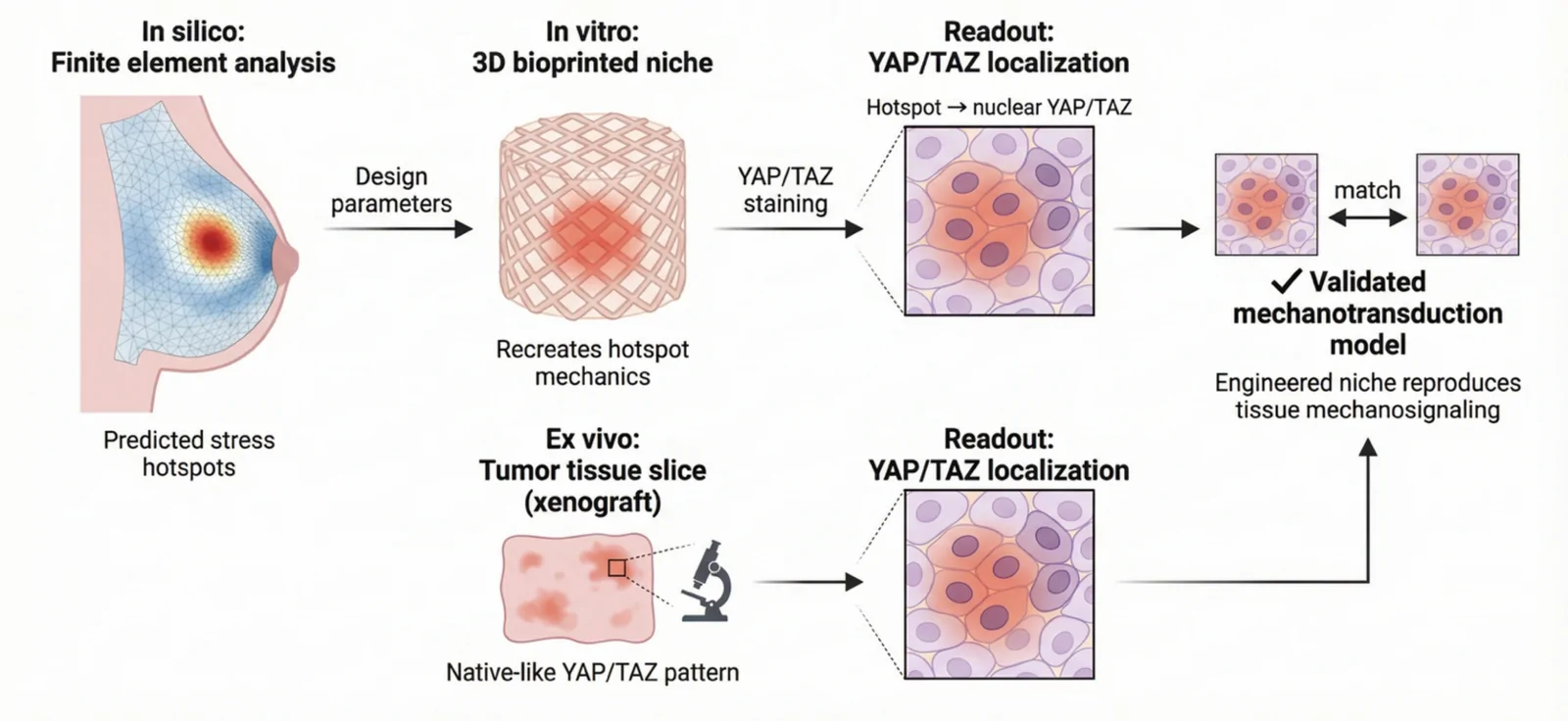
Designing, building, and validating preclinical models of mechanotransduction in breast cancer. Schematic of a patient-informed design–build–validate pipeline for breast cancer mechanotransduction modeling. During *Design*, patient-derived mechanical, architectural, cellular, and molecular measurements, together with computational analyses, are translated into explicit model specifications. During *Build*, an appropriate experimental platform, illustrated here by a 3D bioprinted niche, is constructed to reproduce the selected mechanical features. During *Validate*, the engineered model is compared with corresponding *ex vivo* tissue or xenograft at mechanical, architectural, molecular, and functional levels, and discordant outputs are used to refine the next design iteration. YAP/TAZ localization is shown as one example readout; any quantitative mechanobiological signal or outcome appropriate to the study question may be used. The illustrated workflow is primarily patient-specific, although repeated application across well-annotated cohorts could contribute to shared reference ranges or an atlas of recurrent biomechanical niches. Figure created with BioRender

The Design–Build–Validate pipeline illustrated in Fig. 3 is offered as a workflow for organizing these complementary approaches. During *Design*, investigators should define the clinical or mechanistic question and identify the patient or tissue context to be modelled. Relevant inputs may include local stiffness and viscoelasticity, collagen organization, ligand presentation, solid and interstitial stresses, oxygen tension, cellular composition, and spatially matched molecular readouts. Patient-derived measurements, imaging, and computational analyses can then translate these observations into explicit specifications for the magnitude, distribution, and duration of the mechanical cues of interest. During *Build*, the platform should be selected or combined according to those specifications. A tunable matrix or organoid may be appropriate for local invasion and remodeling; a bioreactor or vascularized chip may be required for dynamic loading or vascular events; bioprinting may be useful when spatial organization is central; and *in vivo* models may be needed when intact stromal, vascular, or metastatic contexts are indispensable. The bioprinted niche shown in Fig. 3 is therefore illustrative, rather than prescriptive.

During *validation,* model outputs should be compared with appropriate patient-derived, *ex vivo*, or *in vivo* benchmarks. Engineering and mechanical validation should establish that the intended force, flow, deformation, stiffness, or stress metric was achieved at the relevant spatial scale. Architectural validation should assess ECM composition, collagen architecture, porosity, vascular organization, and cell distribution. Molecular validation should examine mechanotransductive pathways relevant to the question, and functional validation should determine whether the model accurately reproduces invasion, metastatic behaviour, immune responses, or therapeutic sensitivity. YAP/TAZ localization is used in Fig. 3 as one measurable example; MRTF/SRF activity, Piezo-dependent responses, cytoskeletal or nuclear morphology, ECM remodeling, and treatment response may be equally appropriate. Discordance between the engineered and reference systems should inform iterative redesign. Material properties, applied forces, cell sources, dimensionality, and culture conditions should therefore be measured and reported rather than inferred from nominal design settings.

As presented, this workflow is primarily a patient-informed model development strategy, rather than a clinical treatment selection algorithm. Mechanical and molecular measurements from an individual tumor could inform construction and evaluation of a corresponding model under patient-matched conditions. Repeated application across well-annotated ancestry cohorts could also support a shared atlas of recurrent mechanical niches, reference ranges, and model design parameters for use across institutions and populations. Such an extension would require standardized reporting and careful distinction among genetically-inferred ancestry, self-reported race or ethnicity, tumor subtype, mammographic density, and social or environmental context such that population-level patterns are not overgeneralized. Ultimately, future progress will depend on linking mechanistic depth with reproducibility and clinical relevance. Rigorously benchmarked, ancestry-informed, and multiscale systems may then help resolve how biomechanical context alters metastatic potential or therapeutic response. Treating *Design*, *Build*, and *Validate* as an iterative process therefore offers a practical framework for advancing mechanobiology-informed breast cancer research while defining the scope and limitations of each model.

Thus, advancing an ancestry- and mechanobiology-informed framework in breast cancer research and modeling will be essential for the viability of future translational outcomes. This unified approach is necessary and particularly timely as the United States Food and Drug Administration and other international regulatory agencies migrate away from interpreting monolayer cell culture and large-scale animal studies alone as sufficient predictions of patient pathology and outcomes. This framework offers a more comprehensive toolkit for elucidating the roles of mechanotransduction in breast cancer emergence and proliferation, and holds promise for informing personalized therapeutic strategies and reducing disparities in clinical outcomes originating from biomechanical variability.

## Data Availability

No datasets were generated or analyzed during the current study.

## Declarations

### Clinical trials

Clinical trial number: not applicable.
